# A comparison of TURP, HoLEP, and RFA for nonneurogenic LUTS in men

**DOI:** 10.55730/1300-0144.5979

**Published:** 2024-12-20

**Authors:** Turgay EBİLOĞLU, Özgür ÇINAR, Selçuk SARIKAYA, Adem Emrah COĞUPLUGİL, Bahadır TOPUZ, Cengiz KARA, Selahattin BEDİR

**Affiliations:** Department of Urology, Gülhane Training and Research Hospital, Ankara, Turkiye

**Keywords:** TURP, HoLEP, RFA, LUTS

## Abstract

**Background/aim:**

The effect of transurethral resection of the prostate (TURP) for nonneurogenic male lower urinary tract symptoms (LUTS) is well known. However, recent advancements have come into use, so studies have been done to compare these new techniques to the gold standard TURP technique. The aim of this study was to compare the results of TURP, holmium laser enucleation of the prostate (HoLEP), and radiofrequency ablation (RFA) in men.

**Materials and methods:**

The patients who had the TURP procedure were defined as group 1 (G1), those who had HoLEP were in group 2 (G2), and those who had RFA were in group 3 (G3). Preoperative and postoperative results were compared, with postoperative checks done at 1, 12, 24, and 36 months.

**Results:**

There were 41, 40, and 40 patients in G1, G2, and G3, respectively. The mean ages for G1, G2, and G3 were 68.21 ± 8.19, 65.44 ± 10.48, and 77.32 ± 10.58, respectively. The decrease in international prostate symptom score (IPSS) was similar in G1 and G2 for all follow-up periods, but the decrease for G3 was smaller. The quality of life improvement can be summarized as G1 > G2 > G3 at the 36-month follow-up. Scores on the overactive bladder questionnaire (OAB-V8) decreased at the 1-month follow-up for G1 and G2, but then started to increase again over time. Scores on the international index of erectile function (IIEF-5) improved in a continuous fashion up to the 36^th^ postoperative month for G1. The incontinence rate was highest in G2 for all time periods despite a decrease after the first postoperative month. The incontinence rate was the lowest for G3 across all time periods.

**Conclusions:**

The TURP and HoLEP procedures yielded equal improvement in IPSS, however HoLEP had a higher incontinence rate. RFA did not yield much improvement in IPSS, however it seems suitable for older patients with the lowest incontinence rates. None of the techniques provided an improvement in terms of the OAB-V8.

## 1. Introduction

Nonneurogenic male lower urinary tract symptoms (LUTS) due to benign prostatic obstruction (BPO) is a common reason for urinary discomfort in older men. This situation occurs in one third of men over 60 years old [1] The European Association of Urology guideline recommends surgery for BPO when patients have urinary retention, recurrent urinary infections, bladder stones or diverticula, resistant hematuria, or symptoms not responding to behavioral and medical treatments. The most commonly reported indication for surgery is nonresponsive symptoms.

Transurethral resection of the prostate (TURP), developed by Stern in 1926, is the gold standard surgery for BPO, with a 85%–90% success rate [2–4]. In 1983, Hiraoka first described the procedure of endoscopic enucleation of the prostate with a monopolar instrument [5], and in 1998, Fraundorfer and Gilling introduced the enucleation technique using a holmium laser fiber, which is now called holmium laser enucleation of the prostate (HoLEP) [6]. In 1993, a less invasive thermal ablation technique evolved based on radiofrequency ablation (RFA). RFA was intended as an outpatient procedure and advised for use with high-risk patients as it did not require spinal or general anesthesia [7,8].

Many studies have been done on the success and complications of TURP, HoLEP and RFA, but as of yet no study has compared the effects of TURP, HoLEP, and RFA in terms of uroflowmetry and postvoid residual urine (UF-PVR), pathologic results, international prostate symptom score (IPSS) + quality of life (QoL), overactive bladder questionnaire results (OAB-V8), international index of erectile function (IIEF-5) score, total prostate-specific antigens (PSA), and stress incontinence rates up to 36 months postoperatively. The aim of this study is to compare the 36-month results of these three techniques using a sample of Turkish male patients.

## 2. Materials and methods

This study was approved by the Ethical Committee of Gülhane Training and Research Hospital with number 2023-225) and was conducted following the institution’s human subject guidelines. All patients gave their signed informed consent for participation in the study.

Between October 2016 and January 2024, a total of 121 patients with a small prostate volume (≤95 cc) needing prostate surgery for nonneurogenic male LUTS were enrolled in this retrospective study. The patients were compared based on whether they had the TURP, HoLEP, or RFA procedure.

A thorough history was taken including voiding habit and constitutional urologic abnormalities. All patients underwent a complete physical examination, including an inspection of the external urethral meatus. After the physical examination, all patients were evaluated by serum total PSA and urinary ultrasonography. The patients were also asked to fill out the IPSS + QoL, OAB-V8, and IIEF-5 questionnaires. The UF-PVR checks were performed by a trained nurse using an MMS 5000 urodynamic device and BladderScan BVI 6100 (Diagnostic Ultrasound, Bothell, WA, USA) at the hospital’s urodynamic laboratory.

After the operations, the patients were checked at 1, 12, 24, and 36 months postoperatively. At the 1-month check, UF-PVR, pathologic results, IPSS + QoL, OAB-V8, IIEF-5, total PSA, and stress incontinence rates were recorded. At the following checks, all the same parameters were checked except for the pathologic results and UF-PVR.

The inclusion criteria for deciding to use a surgical procedure were having recurrent urinary retention, recurrent urinary tract infections, bladder stones, dilatation of the upper urinary tract due to prostatic obstruction, resistant hematuria from the prostate, and refractory symptoms despite trying at least three different medical regimens, including alpha blockers, 5 alpha reductase inhibitors, anticholinergics, beta3 agonists, PDE5 inhibitors, or a combination of these protocols. The exclusion criteria for RFA were a prostatic urethral length outside the range of 23–35 mm and an evident middle lobe. The prostatic urethral length was measured by transrectal ultrasound.

Once the decision to use a surgical intervention was made, the patients were checked for anesthesia suitability by physicians in that department. Patients deemed unsuitable for spinal or general anesthesia were given the RFA procedure. If anesthesia was possible, they were randomly given either TURP or HoLEP operations. The TURP patients were defined as group 1 (G1), the HoLEP patients were defined as group 2 (G2), and the RFA patients were defined as group 3 (G3).

### 2.1. Transurethral resection of the prostate (TURP)

After spinal or general anesthesia, the patients were positioned in the lithotomy position. A 26 Fr bipolar resectoscope was used for the operation (Olympus, Tokyo, Japan). Using the Nesbit technique, first the median lobe was resected, and then the lateral lobes were resected up to the prostate capsule.

### 2.2. Holmium laser enucleation of the prostate (HoLEP)

After spinal or general anesthesia, the patients were positioned in the lithotomy position. A 26 Fr HoLEP endoscope was used for the operations. Using the trilober technique, first the median lobe and then the lateral lobes were enucleated from the prostate capsule. A 100-watt laser was used (Lumenis Holmium Laser System, Boston Scientific, USA). After the enucleation, the separated lobes were morcellated with special attention paid to the bladder mucosa.

### 2.3. Radiofrequency ablation (RFA)

The patients were positioned in a supine position. After application of a 2% lidocaine local anesthetic lumbricant agent, a specific 16F urethral catheter with six electrodes at the tip was inserted from the urethra, similar to a routine urethral catheter application. The electrodes were attached to the RF unit (Tempro: Initia Ltd., Direx Group) and heated to 55 °C. The procedure typically took 1 h.

### 2.4. Statistical analysis

Statistical analysis was done using SPSS v.26.0 (SPSS, Chicago, USA) by an expert biomedical statistician. A power analysis was done by using the G power analyzer, determining that 40–45 patients were sufficient for each of the three comparison groups. Descriptive statistics were noted with mean ± standard deviation (minimum–maximum), numbers, and percentiles. The Kolmogorov–Smirnov test was used to assess variable normalization. The independent sample t-test was used to compare the preoperative and postoperative independent scale parameters with normal distribution. The Mann–Whitney U Test was used to compare the preoperative and postoperative independent scale parameters without a normal distribution. The paired sample t-test was used to compare the preoperative and postoperative dependent scale parameters with a normal distribution. The Wilcoxon test was used to compare the preoperative and postoperative dependent scale parameters without a normal distribution. The McNemar test was used to compare the preoperative and postoperative dependent nominal parameters. The chi-square test was used to compare the preoperative and postoperative independent nominal parameters. The repeated measures analysis of variance test was used to compare the scale related and repeated measures with a normal distribution. The Friedman test was used to compare the scale related and repeated measures without a normal distribution. A probability of p < 0.05 was accepted as statistically significant.

## 3. Results

A total of 600 patients had procedures for BPO, but only patients who fully completed the visits and follow-up parameters were included in the study. There were 41, 40, and 40 patients in groups G1, G2, and G3, respectively, with corresponding mean ages of 68.21 ± 8.19, 65.44 ± 10.48, and 77.32 ± 10.58 (p = 0.16 between G1 and G2, p = 0.002 between G1 and G3, and p = 0.001 between G2 and G3). There were no statistically significant differences in preoperative main symptoms, alpha blocker use, urinary retention rate, OAB-V8 score, IEFF-5 score, total PSA, prostate volume, and bladder stone rate among the groups (Table 1). However, preoperative IPSS and QoL scores were lower in G3 than the other groups despite not being statistically significant (p = 0.452 and p = 0.289, respectively) (Table 1).

The preoperative and first postoperative month UF maximum flow rates (Qmax) (mL/s), average flow rates (mL/s), and PVR volumes (cc) are shown in Table 2. The preoperative to postoperative changes in UF-PVR were significant and similar for G1 and G2. However, there were no significant changes for G3.

The postoperative pathology results yielded one case of prostate cancer in G1 and three cases in G2 (p = 0.269).

Comparisons of the preoperative and all the postoperative follow-up results are shown in the Figure. According to these results, the IPSS scores for all groups decreased significantly postoperatively until the 12th month (p = 0.008); however, afterwards they significantly increased (p = 0.002) for all groups. The IPSS change was similar for G1 and G2 (p = 0.625) at all follow-up intervals. However, the IPSS drop was not as dramatic for G3 as for the other groups (p = 0.003 between G1 and G3 and p = 0.002 between G2 and G3). Comparing the preoperative results with the 36-month postoperative results, the IPSS drop was still statistically significant for G1 and G2 (p = 0.0001 for both), but not for G3 (p = 0.817) (Figure).

The QoL score was parallel to the IPSS change for G1 and G2 in that it decreased significantly in the first 12 months before starting to increase again. For G3, QoL decreased significantly at the 1-month follow-up, and then it started to increase. Patients in G1 had the best QoL after their operations, but the difference was not statistically significant from the other groups (p = 0.109) at the 1-month follow-up. However, starting at the 12-month follow up, G1 and G2 had better improvement than G3 (p = 0.004 between G1 and G3 and p = 0.001 between G2 and G3), and this status continued until the end of the monitoring. Overall, the QoL improvement could be summarized as G1 > G2 > G3 at the 36-month follow-up (p = 0.125 between G1 and G2, p = 0.04 between G1 and G3, and p = 0.02 between G2 and G3) (Figure).

For G1 and G2, the OAB-V8 score decreased at the 1-month follow-up (p = 0.04) but then started to increase so that by the 36th month, the improvement was not significantly different from the preoperative status (p = 0.878). For G3, there was no improvement at any of the follow-up intervals; in fact, there was deterioration (Figure).

The IIEF-5 score improved in a continuous fashion up to the 36th month for G1. The change was not statically significant between the follow-up intervals, but it reached a statistically significant level at the 36-month follow-up compared to the preoperative status (p = 0.015). G2 and G3 both had a decline in IIEF-5 score, but the decline was worse for G3 than G2 (p = 0.482) (Figure).

The PSA decrease was significant for all groups at the 1-month follow-up (p = 0.032), and then PSA stayed largely stable for G1 and G2 up to the 36-month follow-up with some intermittent increases. For G3, PSA started to increase after the first follow-up and became higher than the preoperative level at the 24-month follow-up (p = 0.012) (Figure).

Of the three groups, the stress incontinence rate was the highest for G2 at the first follow-up. It decreased at the later follow-ups but stayed higher than the other groups for all intervals (p = 0.002 between G2 and G1 and p = 0.003 between G2 and G3). In G1, the incontinence rate increased at the 12-month follow-up and remained that way without any significant decrease. G3 had the lowest incontinence rate for all intervals (p = 0.002 between G2 and G1 and p = 0.003 between G2 and G3) (Figure).

## 4. Discussion

The main goal of BPO surgery is to achieve the most effective results with the most noninvasive technique and the lowest complication rates. With this objective, various technological improvements have been adopted for BPO surgery over the years. First endoscopic instruments were used, then ablation technology, and next laser technology developed. Historically, TURP, endoscopic enucleation with monopolar instruments, RFA, and HoLEP were introduced in 1926, 1983, 1993, and 1998, respectively. Rationally, the success parameters and the complications of any technique must be explained in the same standardized way, but the literature on both aspects of these techniques is scarce. Some studies have only focused on the success parameters while others have focused only on the complications. To simplify comparison, the success results of the measured outcomes of IPSS, QoL, Qmax, and PVR are presented here in a standard way.

The oldest, and gold standard, method for BPO surgery is TURP. Reich et al. reported an increase in urinary Qmax to 21.6 mL/s and a decrease in PVR to 31.6 cc shortly after surgery [9]. Yoon et al. reported preoperative and 1-, 6-, and 12-month postoperative results for IPSS and Qmax, with IPSS values of 18.7, 6.6, 6.5, and 6.7 and Qmax values of 8.7, 17.4, 18.9, and 18.8 mL/s, respectively [10]. Erturhan et al. reported preoperative and 1- and 12-month postoperative results for IPSS, QoL, Qmax, and PVR. Respectively for the three reported intervals, their IPSS values were 24, 5, and 4, QoL values were 3, 2, and 2, Qmax values were 7, 26, and 18 mL/s, and PVR values were 135, --, and 25 cc [11]. De Sio et al. reported preoperative and 3-, 6-, and 12-month postoperative results for IPSS, QoL, Qmax, and PVR for TURP. Respectively for the four reported intervals, their IPSS values were 20, 8, 5, and 3, QoL values were 4, 1.3, 1, and 0.8; Qmax values were 6, 21, 20 and 20 mL/s, and PVR values were 80, 41, 38, and 22 cc [12].

Enucleation with monopolar instruments evolved after TURP, but the addition of laser technology into the enucleation procedures delayed the common use of this technique until the 2000s. The difficulty of the enucleation technique is the steep learning curve, with 25 cases needed to learn the technique and 50 for mastery being reported [13]. The HoLEP technique is commonly believed to be superior to TURP for all success and complication outcomes covered above. In a systemic review by Chen et al. comparing TURP and HoLEP, HoLEP had shorter operation times, shorter catheterization times, shorter hospitalization times, more resected tissue, reduced hemoglobin decreases, greater PVR decreases, and a similar QoL to TURP [14]. Lee et al. reported preoperative and 3- and 6-month postoperative results for IPSS, QoL, Qmax, and PVR in 3000 HoLEP patients. Respectively for the three intervals, their IPSS values were 19, 8, and 7, QoL values were 3.9, 1.8, and 1.4 Qmax values were was 9.4, 21.8, and 22.2 mL/s, and PVR values were 51, 6, and 2 cc [15]. Gilling et al. reported preoperative and 1-, 3-, 6-,12 -, and 72-month postoperative results for IPSS, QoL, and Qmax in 71 patients. Respectively for all six intervals, their IPSS values were 25.7, 9.7, 7.9, 7.5, 6.6, and 8.5, QoL values were 4.9, 2.7, 1.9, 1.7, 1.6, and 1.8, and Qmax values were 8.1, 20.3, 20.7, 23, 20.9, and 19 mL/s [16].

In 1993, RFA evolved for the treatment of BPO. Despite the early introduction of the technique, it has not been commonly used worldwide [7,8]. Diri et al. reported preoperative and 6-month postoperative results for IPSS, QoL, Qmax, and PVR. Respectively for the two intervals, their IPSS values were 21.15 and 13.07, QoL values were 4.51 and 2.61, Qmax values were 5.97 and 10.61 mL/s, and PVR values were 25.42 and 23.18 cc [7]. Engin et al. reported preoperative and 1-, 3-, 6-, 12-, and 24-month postoperative results for IPSS, QoL, Qmax, and PVR. Respectively for the six intervals, their IPSS values were 23, 10, 7, 7, 7, and 6, QoL values were 4.5, 2.7, 1.7, 1.3, 1.3, and 1.3, Qmax values were 10, 12, 14, 14, 14, and 15 mL/s, and PVR values were 57, 41, 33, 31, 30, and 29 cc [8].

A comparison of the results of this study with those in the literature shows that they are comparable, including the reported IPSS and QoL improvements. In this study, these parameters were reported until 36 months postoperatively, whereas the studies in the literature mostly report until the 12th postoperative month. In this study, IPSS decreased across all groups, but the greatest decrease was in the HoLEP group. On the other hand, the overall preoperative and postoperative IPSS scores were higher in this study than in the literature, which indicates our study included cases with higher severity. Nevertheless, the reduction in IPSS in this study was similar to the literature.

TURP had the largest QoL decrease, HoLEP was in the middle, and RFA had the smallest. RFA also had the smallest IPSS decrease, and HoLEP had the largest. The Qmax increase from preoperative to 1-month postoperative was 10.75 to 35.26 mL/s for TURP, 10.43 to 27.74 mL/s for HoLEP, and 11.07 to 14.79 mL/s for RFA. The PVR decrease from preoperative to 1-month postoperative was 145 to 21.3 for in TURP, 179.8 to 30.79 cc for HoLEP, and 141.42 to 118.5 cc for RFA.

Concerning complications, a large review article by Mebust et al. on TURP reported 2.5% bleeding needing transfusion, 2% TUR syndrome, 1.1% myocardial arrhythmia, 0.9% urinary extravasation in intraoperative course, 6.5% voiding failure, 3.9% bleeding needing transfusion, 3.3% clot retention, and 2.3% urinary infection in the early postoperative period [17]. Another large review article by Rassweiler et al. reported more late postoperative complications. They reported 30%–40% early urge incontinence and 0.5% late stress incontinence, 2.2%–9.8% urethral stricture without any correlation to the postoperative time period, 0.3% bladder neck stricture, 53%–75% retrograde ejaculation, 3.4%–32% erectile dysfunction, and 3%–14.5% recurrent BPO up to 5 years postoperatively [18]. For HoLEP, Chen et al. reported stress and urge incontinence rates of 5% and 3.4%, respectively, in the third postoperative month and 1.8% and 0.9% in the sixth postoperative month, respectively [14]. Castenada et al. reported a 35% incontinence rate consisting of 20.8% urge and 12.7% stress incontinence in the first month after HoLEP but also reported a decrease to lower levels in later months [19]. In contrast, Akbal et al. reported no stress or urge incontinence at 6 and 12 months postoperatively [20]. Gilling et al. reported average erectile function and overall satisfaction six years after HoLEP [16]. For RFA, Diri et al. reported an IIEF-5 increase at the sixth postoperative month [7].

The literature includes no discussion of overactive bladder symptoms and sexual status, and no studies mentioned incontinence rates or overactive bladder symptoms after RFA. To fill this gap, this study focused on results for IPSS, QoL, OAB-V8, IIEF-5, PSA, and stress incontinence rates with comparisons among the techniques. OAB-V8 is commonly used to detect overactive bladder symptoms, and IIEF-5 is commonly used to detect sexual status for men.

Four differentiating points have come out of this study. The primary difference of this study from others was in comparing the preoperative and postoperative overactive bladder symptoms after different procedures. It was observed that none of the techniques provided significant improvement in OAB-V8, but the best technique was TURP and the worst was RFA, which in fact caused an increase in OAB-V8 symptoms. The second difference of this study was comparing the preoperative and postoperative sexual status of the different procedures using the IIEF-5. The best technique for sexual health improvement was determined to be TURP, which goes contrary to the previous results in the literature. The reason for this could be due to using the IIEF-5 scoring system, which consists mostly of questions for erectile function rather than ejaculation function. Only the last question may be related to ejaculation function, but this is not sufficient to properly evaluate sexual health. It seems that TURP results in the best erectile function, but the same may not be true for ejaculation. The other techniques were determined to score lower on IIEF-5, with RFA being the worst concerning sexual status using IIEF-5. PSA change was the third difference in this study. All of the techniques decreased PSA postoperatively; TURP and HoLEP had similar and significant decreases, but RFA caused only a small decrease. The fourth difference was in the comparison of stress incontinence rates up to the 36th postoperative month. The highest stress incontinence rate was in the HoLEP technique, reaching 80% at the first postoperative check and decreasing to 20% at the last one.

The primary limitation of this study was that the G3 patients were older than those in the other groups. Despite the power analysis results, another limitation was having a relatively small sample size. The third limitation was not using a prospective approach. Although the patients were randomly assigned to G1 or G2, the fact that it was not a prospectively planned study meant that getting correct urethral stricture and retrograde ejaculation results was not possible, so these parameters could not be reported.

## 5. Conclusion

TURP and HoLEP yielded equal improvement in IPSS and UF-PVR, however HoLEP had a higher incontinence rate. RFA did not yield much improvement in IPSS and UF-PVR, however it seems suitable for older patients with the lowest incontinence rates. None of the techniques provided an improvement in OAB-V8.

## Figures and Tables

**Figure:**
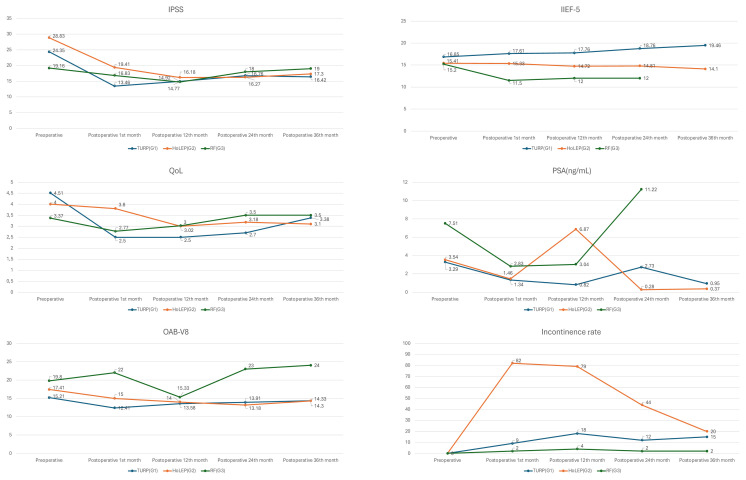
Preoperative and 1-, 12-, 24-, and 36-month postoperative IPSS, QoL, OAB-V8, IIEF-5, PSA, and stress incontinence values

**Table 1:** Preoperative demographic parameters of all groups.

	*Urination difficulty*	*Frequency*	*Nocturia*	*p*
TURP(G1)	6	3	3	*0.890*
HoLEP(G2)	3	6	2	
RFA(G3)	3	6	2	
*Previous alpha blocker*				
TURP(G1)	29			*0.650*
HoLEP(G2)	26			
RFA(G3)	38			
*Previous urinary retention*				
TURP(G1)	2			*0.682*
HoLEP(G2)	3			
RFA(G3)	14			
*Previous IPSS score*				
TURP(G1)	24.35±6.53 (12–33)			*0.452*
HoLEP(G2)	23.83±5.16 (14–32)			
RFA(G3)	19.16±5.89 (10–32)			
*Previous quality of life score*				
TURP(G1)	4.5±1.28 (2–6)			*0.289*
HoLEP(G2)	4±1.04 (2–6)			
RFA(G3)	3.37±1.18 (2–6)			
*Previous OAB-8 score*				
TURP(G1)	15.21±5.78 (10–28)			*0.487*
HoLEP(G2)	17.41±4.16 (10–23)			
RFA(G3)	19.8±5.31 (15–28)			
*Previous IEFF-5 score*				
TURP(G1)	16.85±6.44 (6–25)			*0.653*
HoLEP(G2)	15.41±6.35 (5–25)			
RFA(G3)	15.2±9.65 (5–25)			
*Previous total PSA*				
TURP(G1)	3.29±2.21 (0.58–8.26)			*0.742*
HoLEP(G2)	3.54±4.59 (0.25–18.92)			
RFA(G3)	7.51±5.42 (0.32–25.3)			
*Prostate volume*				
TURP(G1)	63.54±18.57 (37–91)			*0.243*
HoLEP(G2)	55.79±16.05 (27–80)			
RFA(G3)	53.77±20.5 (17–95)			
*Previous bladder stone*				
TURP(G1)	2			*0.064*
HoLEP(G2)	4			
RFA(G3)	0			

**Table 2:** The preoperative and postoperative UF-PVR parameters.

		TURP (G1)	HoLEP (G2)	RFA (G3)
*Maximum flow rate(mL/s)*	Preoperative	10.75±4.53 (6–15)	10.43±3.12 (4–13)	11.07±6.65 (3.4–12)
	Postoperative 1st month	35.26±12.33 (22–50)	27.74±11.22 (16–42)	14.79±8.09 (3.2–32)
*Average flow rate(mL/s)*	Preoperative	6.41±3.12 (3–9)	6.92±2.41 (5–11)	4.87±3.73 (1–12)
	Postoperative 1st month	16.9±5.32 (11–20)	15.18±5.33 (10–21)	6.05±3.47 (1–14)
*Post void residue (PVR)(mL)*	Preoperative	145±75 (62–500)	179.8±150.79 (51–479)	141.72±102.1 (0–500)
	Postoperative 1st month	21.3±8.24 (0–42)	30.79±9.78 (0–47)	118.5±93.36 (0–400)

TURP and HoLEP had a significant effect on UF-PVR (p < 0.05), but RFA had no significant effect (p > 0.05).

## References

[1] Cornu JN Gacci M Hashim H Herrmann TRW Malde S EAU 2024 Guidelines Cornu JN EAU Guidelines on Non-Neurogenic Male Lower Urinary Tract Symptoms (LUTS)

[2] Wasson JH Reda DJ Bruskewitz RC Elinson J Keller AM A comparison of transurethral surgery with watchful waiting for moderate symptoms of benign prostatic hyperplasia New England Journal of Medicine 1995 332 2 75 79 10.1056/nejm199501123320202 7527493

[3] Holtgrewe HL Mebust WK Dowd JB Cockett ATK Peters PC Transurethral prostatectomy: practice aspects of the dominant operation in American urology Journal of Urology 1989 141 2 248 253 10.1016/s0022-5347(17)40732-4 2643720

[4] Strebel RT Kaplan SA The state of TURP through a historical lens World Journal of Urology 2021 39 7 2255 2262 10.1007/s00345-021-03607-7 33772604

[5] Hiraoka Y A new method of prostatectomy, transurethral detachment and resection of benign prostatic hyperplasia Journal of Nippon Medical School 1983 50 6 896 10.1272/jnms1923.50.896 6199367

[6] Fraundorfer MR Gilling PJ Holmium: YAG laser enucleation of the prostate combined with mechanical morcellation: preliminary results European Urology 1998 33 1 69 72 10.1159/000019535 9471043

[7] Diri MA Gul M Effect of bipolar radiofrequency thermotherapy on benign prostate hyperplasia Andrologia 2020 52 2 e13467 10.1111/and.13467 31692009

[8] Engin Ö Remzi S Ferat ÖH Our experiences in bipolar radiofrequency thermotherapy system as an alternative treatment in LUTS LUTS: Lower Urinary Tract Symptoms 2020 12 3 235 239 10.1111/luts.12309 32227470

[9] Reich O Gratzke C Bachmann A Seitz M Schlenker B Morbidity, mortality and early outcome of transurethral resection of the prostate: a prospective multicenter evaluation of 10,654 patients Journal of Urology 2008 180 1 246 249 10.1016/j.juro.2008.03.058 18499179

[10] Yoon CJ Kim JY Moon KH Jung HC Park TC Transurethral resection of the prostate with a bipolar tissue management system compared to conventional monopolar resectoscope: one-year outcome Yonsei Medical Journal 2006 47 5 715 720 10.3349/ymj.2006.47.5.715 17066516 PMC2687758

[11] Erturhan S Erbagci A Seckiner I Yagci F Ustun A Plasmakinetic resection of the prostate versus standard transurethral resection of the prostate: a prospective randomized trial with 1-year follow-up Prostate Cancer and Prostatic Diseases 2007 10 1 97 100 10.1038/sj.pcan.4500907 16926854

[12] Sio MD Autorino R Quarto G Damiano R Perdona S Gyrus bipolar versus standard monopolar transurethral resection of the prostate: a randomized prospective trial Urology 2006 67 1 69 72 10.1016/j.urology.2005.07.033 16413335

[13] Kampantis S Dimopoulos P Tasleem A Acher P Gordon K Assessing the learning curve of holmium laser enucleation of prostate Urology 2018 120 9 22 10.1016/j.urology.2018.06.012 30403609

[14] Chen J Dong W Gao X Li X Cheng Z A systematic review and meta-analysis of efficacy and safety comparing holmium laser enucleation of the prostate with transurethral resection of the prostate for patients with prostate volume less than 100 mL or 100 g Translational Andrology and Urology 2022 11 4 407 420 10.21037/tau-21-1005 35558272 PMC9085931

[15] Lee H So S Cho MC Cho SY Paick JS Clinical outcomes of holmium laser enucleation of the prostate: a large prospective registry-based patient cohort study under regular follow-up protocol Investigative and Clinical Urology 2024 65 4 361 367 10.4111/icu.20240080 38978216 PMC11231663

[16] Gilling PJ Kennett K Das AK Thompson D Fraundor MR Holmium laser enucleation of the prostate (HoLEP) combined with transurethral tissue morcellation: an update on the early clinical experience Journal of Endourology 1998 12 5 457 459 10.1089/end.1998.12.457 9847070

[17] Mebust WK Holtgrewe HL Cockett ATK Peters PC Transurethral prostatectomy: immediate and postoperative complications. A cooperative study of 13 participating institutions evaluating 3,885 patients. 1989 Journal of Urology 2002 167 2 Pt 2 999 1003 11908420

[18] Rassweiler J Teber D Kuntz R Hofmann R Puppo P Complications of transurethral resection of the prostate (TURP)—incidence, management, and prevention European Urology 2006 50 5 969 980 10.1016/j.eururo.2005.12.042 16469429

[19] Agreda-Castañeda F Freixa-Sala R Franco M Bultó-Gonzalvo R Areal-Calama J Predictive factors of post-HoLEP incontinence: differences between stress and urgency urinary incontinence World Journal of Urology 2024 42 1 281 10.1007/s00345-024-04984-5 38695948

[20] Aybal HC Yilmaz M Barlas IS Duvarci M Tuncel A Comparison of HoLEP, ThuLEP and ThuFLEP in the treatment of benign prostatic obstruction: a propensity score-matched analysis World Journal of Urology 2024 42 1 374 10.1007/s00345-024-05082-2 38871959

